# Presence of Varicella Zoster Virus DNA in Saliva May Be Associated with the Severity of Ramsay Hunt Syndrome

**DOI:** 10.3390/biomedicines10092177

**Published:** 2022-09-02

**Authors:** Gang Won Choi, Junyang Jung, Seung Geun Yeo, Sang Hoon Kim

**Affiliations:** 1 Department of Otorhinolaryngology, Head and Neck Surgery, School of Medicine, Kyung Hee University, Seoul 02453, Korea; 2 Department of Anatomy, School of Medicine, Kyung Hee University, Seoul 02453, Korea

**Keywords:** Ramsay Hunt syndrome, varicella zoster virus, DNA in saliva, electroneurography, facial palsy

## Abstract

Background: The relationship between varicella zoster virus (VZV) collected from saliva and Ramsay Hunt syndrome (RHS) remains unclear. Therefore, this study aimed to investigate whether VZV DNA in saliva alters the clinical symptoms and prognosis of RHS. Methods: To measure the severity of clinical symptoms of 100 RHS patients, the initial House–Brackmann (HB) grade and associated symptoms were evaluated. The final HB grade at the end of treatment was measured to determine the prognosis. Electroneurography (ENoG) was performed on four facial muscles, including the frontalis, oculi, nasalis, and oris. Results: Salivary VZV DNA was isolated from 72 patients with RHS. The VZV DNA-positive group was 34.023 times more likely to have severe initial HB grade than the control group (95% CI, 3.21–359.68; *p* = 0.003). There were no significant differences in final HB grade. All ENoG values of the VZV DNA-positive group were significantly higher than those of the control group (frontalis, *p* = 0.003; oculi, *p* = 0.001; nasalis, *p* < 0.001; oris, *p* = 0.037). Conclusions: RHS patients with salivary VZV DNA have more severe clinical symptoms than the control group. There were no differences in prognosis and associated symptoms. A VZV DNA test using saliva samples of patients with RHS can evaluate the clinical symptoms and provide early confirmation of VZV infection, enabling timely treatment in a non-invasive way.

## 1. Introduction

Ramsay Hunt syndrome (RHS) is a neurological disease caused by the varicella zoster virus (VZV) and is the second most common cause of acute facial palsy. RHS shows clinical symptoms of vesicles in the ears or oropharynx, accompanied by facial palsy [1]. In some cases, inflammation of the facial nerve spreads to the vestibulocochlear nerve, causing dizziness or hearing disturbance [2]. However, despite facial palsy caused by VZV infection, vesicular lesions may not appear on the ears or may occur a few days after facial palsy, which delays the diagnosis of RHS or zoster sine herpete (ZSH) [3,4]. Moreover, it can be misdiagnosed as Bell’s palsy, and antiviral treatment may be delayed until vesicles are visible. Combination therapy with steroid and antiviral treatment within three days after onset is crucial for a good prognosis of facial palsy [5]. Therefore, a test to confirm VZV infection in patients with facial palsy is essential. Serum antigenic tests have traditionally been used to diagnose VZV infection. However, detecting VZV-specific IgG and IgM antibodies by enzyme-linked immunosorbent assay (ELISA) takes more than two weeks to confirm. PCR-based techniques to detect VZV DNA in saliva or tear auricular lesions have been attempted to rapidly identify VZV infection. In previous studies, VZV DNA was detected in the saliva of patients with zoster lesions in the oropharynx as well as those with RHS and zoster lesions only in the auricle [6]. However, qualitative or quantitative analyses of VZV infection other than clinical symptoms, such as facial palsy and vesicles, are not well implemented in RHS diagnosis. Research on the association between VZV DNA in saliva and RHS remains insufficient. Therefore, in this study, we investigated the difference between the clinical symptoms and prognosis of RHS based on the presence of VZV DNA in the saliva.

## 2. Material and Methods

### 2.1. Patients

This retrospective study included 100 patients diagnosed with RHS at Kyunghee Medical Center, Seoul, Korea between January 2015 and October 2021. RHS patients were diagnosed based on clinical symptoms. Clinical symptoms of RHS include a vesicular rash on the ear (herpes zoster oticus) or in the oral mucosa accompanied by acute peripheral facial nerve paralysis. Patient medical records included information regarding sex, age, treatment period, underlying disease, and morbidity. Comorbid symptoms, such as dizziness, tinnitus, hyperacusis, and hearing distance, were also assessed. The House–Brackmann (HB) grading system was used to evaluate the degree of facial palsy [7]. The HB scale was divided into six clinical stages from grade I (normal function in all facial areas) to grade VI (complete paralysis plus gross asymmetry at rest). The HB grade was measured on the day of hospitalization. The follow-up duration was at least six months after facial palsy or until complete recovery. HB grade measured at the last visit was recorded as the final HB grade. An initial HB grade of V or VI was defined as severe HB. Complete recovery was defined as final HB grade I, and satisfactory recovery was defined as final HB grade I or II.

The inclusion criteria were patients hospitalized within seven days of onset and who were not lost to follow-up until the end of treatment. Patients with incomplete medical records, whose cause of facial palsy was malignancy or central lesion, those who were lost to follow-up during treatment, or those who had previous herpes zoster infection were excluded.

All patients who met the inclusion criteria were treated with oral corticosteroids (1 mg/kg/day for 4 days, followed by 6–8 days of tapering schedule), oral antiviral agents (famciclovir for 1 week), and protective eye care by administration of artificial eye drops or ointment, taping of the paralyzed eyelid during the night, and patient education [8].

### 2.2. Detection of VZV DNA in Saliva by PCR

Saliva samples were collected from the hospitalized patients. The saliva samples were collected by patients themselves spitting 1~2 mL of saliva into the sample container. Samples were collected once on the day of hospitalization. After the samples were collected, these were immediately sent to the molecular genetic testing room and immediately stored in a refrigerator at 4 °C until examination. Viral NA Small Volume Kit (Roche) reagents were used for DNA extraction from saliva samples using the MagNA Pure 96 (Roche). The Pathogen Universal 200 protocol was used for viral DNA isolation from 200 μL of each saliva sample using 100 μL of eluate as per the purification protocol. A low denaturing temperature PCR (LDT-PCR) was performed using conventional PCR reagents and VZV PCR primers. The PCR mixture included 10 μL 2× master mix, 5 μL VZV primers, and 5 μL template. The PCR conditions were 94 °C for 15 min, 94 °C for 30 s, 65 °C for 1 min, 72 °C for 1 min, and 72 °C for 10 min. PCR results were analyzed using electrophoresis and an image analyzer, the Gel Doc XR System (Bio-Rad, Hercules, CA, USA) [9,10]. With the cutoff value criterion of 50, saliva was diagnosed VZV positive when the CT value was 50 or higher.

Venous blood samples of the patients were analyzed using enzyme-linked immunosorbent assay (ELISA). Anti-VZV IgM and IgG titers were measured in the blood samples. Antibody titers of anti-VZV IgM and IgG were interpreted as positive if their ratio was ≥1.10.

### 2.3. Electroneurography

Electroneurography (ENoG) was performed between 5 and 14 days after the onset of facial palsy to evaluate its prognosis using a constant current high-voltage stimulator (model DS7A) by a highly experienced examiner [11,12]. ENoG was performed first on the healthy side and then repeated on the affected side. Supramaximal stimulation of 0.2 ms duration at a rate of 1 Hz was provided by bipolar surface electrodes. A bipolar stimulator was placed on the regions over the temporal, zygomatic, buccal, and truncal branches of the facial nerve and on the skin over the stylomastoid foramen, which innervates or stimulates the frontalis, orbicularis oculi, nasalis, and orbicularis oris, respectively. The electrodes were manually adjusted to determine the best position for generating the compound action potential. For recording, surface electrodes were placed on the upper side of the eyebrow and lateral side of the lower eyelid, nasolabial fold (NLF), and lip. A ground electrode was placed around the patient’s wrist. The ENoG value was defined as the percentage of peak-to-peak amplitudes of the ENoG response on the affected side compared with those on the unaffected side.

### 2.4. Statistical Analysis

Continuous variables, such as ENoG values and age, were compared using *t-*tests. Categorical variables, such as initial and final HB grade, sex, direction, diabetes mellitus (DM), hypertension (HTN), and comorbid symptoms, were compared using chi-squared or Fisher’s exact tests. Multiple regression analysis was performed to determine the effect of VZV DNA in saliva on the initial and final HB grade by controlling confounding variables, such as sex, age, direction, DM, and HTN, and presented as the odds ratio and 95% CI. All statistical analyses were performed using IBM SPSS statistical software. Statistical significance was set at *p* < 0.05.

## 3. Results

A total of 100 patients with RHS were enrolled in this study. The demographical data of the study participants are presented in Table 1. Among these patients, 72 and 28 were VZV DNA-positive and VZV DNA-negative, respectively. The VZV DNA-positive group, including 21 men and 51 women, had an average age of 53.08 ± 13.50 years; 21 showed right facial palsy, and 51 showed left-sided facial palsy. Cranial polyneuropathy, HTN, and DM developed in three, nine, and nine patients, respectively. Otalgia, dizziness, tinnitus, hyperacusis, and hearing disturbance were observed as comorbid symptoms simultaneously with facial palsy in 54, 21, 33, 9, and 23 patients, respectively. In contrast, the VZV DNA-negative group had an average age of 44.85 ± 21.16 years and included 12 men and 16 women. Twelve patients in the group showed right facial palsy, whereas 16 showed left-sided facial palsy. The average treatment period was 10.57 ± 0.90; cranial polyneuropathy, HTN, and DM were observed in 0, 4, and 0 patients, respectively. Otalgia, dizziness, tinnitus, hyperacusis, and hearing disturbance were observed as comorbid symptoms on the same side of the facial palsy in 24, 8, 16, 4, and 5 patients, respectively. There were no significant differences in age, sex, morbidity direction, treatment period, cranial polyneuropathy, HTN, DM, or comorbid symptoms between the two groups.

Table 2 shows the number of patients with RHS with VZV DNA in saliva and VZV IgM in blood serum. A total of 33 patients (33%) were positive for VZV IgM, and 72 were positive for VZV DNA in saliva, indicating a higher efficiency in detecting VZV DNA in saliva. When comparing the number of patients with RHS according to the positive or negative status of VZV IgM and VZV DNA in saliva, 21 of 72 VZV DNA-positive patients (29.5%) were VZV IgM-positive, and 12 of 28 VZV DNA-negative patients (42.8%) were VZV IgM-positive.

Table 3 shows a comparison of the initial HB grade between the VZV DNA-positive and DNA-negative groups. Patients with RHS were divided into two stages according to their initial HB grade. Initial HB grades of II–IV were defined as not severe, and initial HB grades of V and VI were defined as severe. Eighteen patients (25%) in the VZV DNA-positive group had severe initial HB grades, whereas only one patient (3.57%) had severe initial HB grades in the VZV DNA-negative group. These results confirmed that the VZV DNA-positive group had a higher proportion of patients with a severe initial HB grade than the VZV DNA-negative group. There was a significant difference between the two groups (*p* = 0.014); the probability of severe initial HB grade in patients with RHS was 34.023 times higher than that of the negative group (95% CI, 3.21–359.68; *p* = 0.003).

Table 4 shows a comparison between the final HB grades of the VZV DNA-positive and VZV DNA-negative groups. Patients with RHS were divided into two stages according to their final HB grades. Complete recovery was defined as a final HB grade of I. A final HB grade of I or II indicated a satisfactory recovery. There were 33 patients (45.83%) with complete recovery in the VZV DNA-positive group and 16 (57.14%) in the VZV DNA-negative group. The VZV DNA-positive group had a lower proportion of patients with complete recovery than the VZV DNA-negative group; however, there was no significant difference between the two groups. There were 54 patients (75%) with satisfactory recovery in the VZV DNA-positive group and 24 (58.71%) in the VZV DNA-negative group. There were no significant differences between the two groups. 

Table 5 shows the differences in ENoG values measured in the four facial muscles (frontalis, orbicularis oculi, nasalis, and orbicularis oris) depending on VZV DNA positivity or negativity in the saliva. The ENoG values measured in the four facial muscles in the VZV DNA-negative group were 60.41 ± 3.08 (frontalis), 58.66 ± 3.34 (orbicularis oculi), 60.58 ± 3.14 (nasalis), and 74.66 ± 3.05 (orbicularis oris). In contrast, the ENoG values in the VZV DNA-positive group were 78.85 ± 4.92, 80.57 ± 3.38, 83.42 ± 3.54, and 85.71 ± 3.74, respectively. All ENoG values of the VZV DNA-positive group were higher than those of the VZV DNA-negative group. There were significant differences between the two groups (frontalis, *p* = 0.003; orbicularis oculi, *p* = 0.001; nasalis, *p* < 0.001; orbicularis oris, *p* = 0.037).

## 4. Discussion

In this study, we examined differences in clinical and comorbid symptoms as well as prognosis according to the presence or absence of VZV DNA in the saliva of patients with RHS. VZV DNA in saliva was detected in 72% of the patients with RHS, and VZV IgM was detected in 33% of the patients with RHS. The detection efficiency of VZV DNA in saliva was higher than that of VZV IgM in patients with RHS, which confirmed that PCR analysis of VZV DNA in saliva showed a higher diagnosis rate than serological tests in patients with RHS. In previous studies, the detection rates of VZV DNA in the saliva of patients with RHS and ZSH were reported to be between 23 and 63% and 7 and 59%, respectively [6,13,14]. The higher detection rate of VZV DNA in the saliva of patients with RHS in this study might be due to collecting a specific amount of saliva samples from hospitalized patients only and within seven days of the onset (early stage of disease).

The initial HB grade according to the presence or absence of VZV DNA in saliva and the number of patients who showed severe clinical symptoms increased significantly in the VZV DNA-positive group. When comparing the ENoG values in the four facial muscles (frontalis, orbicularis oculi, nasalis, and orbicularis oris) according to the presence or absence of VZV DNA in the saliva of patients with RHS, the VZV DNA-positive group showed higher ENoG values in all four muscles than the control group. This means that the VZV DNA-positive group showed more severe axonal loss than the control group. Thus, detecting VZV DNA in the saliva of patients with RHS is associated with worse clinical symptoms. Moreover, there was no significant difference in the final HB grades categorized as complete recovery and satisfactory recovery, according to the presence or absence of VZV DNA in the saliva. Additionally, comorbid symptoms such as dizziness, tinnitus, otalgia, hyperacusis, and hearing disturbance did not vary significantly depending on the presence or absence of VZV DNA in the saliva. A few studies have shown the association between salivary VZV DNA and RHS. According to one study, the VZV DNA-positive group of patients with facial palsy had a higher final HB grade and no difference in the initial HB grade. Moreover, when compared to the VZV DNA-negative group, the VZV DNA-positive group showed more symptoms of dizziness and hearing disturbance, except otalgia [15]. These findings differ from our study results because the previous study targeted all patients, including those with RHS, Bell’s palsy, and ZSH, whereas our study targeted only patients with RHS. According to another study, VZV DNA in saliva changes according to the onset time of facial palsy and the differences in skin lesions in patients with RHS. Salivary VZV DNA was not detected in patients with zoster infection without facial palsy; however, it was detected in a high amount in patients with zoster infection with facial palsy [16]. Another study reported early detection of VZV DNA in the saliva of patients with ZSH before the anti-VZV antibody level increased, revealing the diagnostic value of this method [17]. Another study reported that repeated detection of VZV DNA in saliva was caused by a zoster infection independent of postherpetic neuralgia [18]. According to Furuta et al., there was no difference in salivary VZV DNA between patients with ZSH and RHS, indicating that VZV formed in geniculate ganglia migrated to the oropharyngeal lesions regardless of VZV forming zoster lesions in the auricular skin [6]. According to another study, patients with RHS with oropharyngeal zoster lesions showed higher VZV DNA content in saliva and a worse prognosis of facial palsy than that of patients with RHS without oropharyngeal zoster lesions [14]. Hence, it is confirmed that active VZV in the oropharyngeal lesions penetrates into saliva and is expressed in the form of VZV DNA, representing the patient’s clinical features.

Continuous inflammation of the facial nerve in the temporal bone and VZV reactivation in the geniculate ganglia cause facial palsy in patients with RHS. The facial nerve is composed of motor, parasympathetic, and sensory fibers. The cell bodies of sensory fibers are in the geniculate ganglion. Some afferent fibers are supplied to the mucous membrane of the oropharynx, external auditory canal, and auricular skin [19]. Reactivated VZV is expressed in auricular and oropharyngeal lesions by navigating through the sensory fibers. According to a previous study, a higher VZV DNA content in saliva and poor prognosis in patients with RHS showing zoster in the oropharyngeal epithelium resulted from the movement of VZV DNA to the oropharyngeal lesions through sensory fibers. Therefore, in this study, we presumed that higher ENoG values in the VZV DNA-positive group indicated extreme nerve damage, resulting in severe clinical features [20].

This study has the advantage of presenting statistical validity by collecting data from a relatively large group of patients. In addition, the ENoG value was measured in the four facial nerves for a more accurate evaluation of clinical symptoms. Despite facial palsy being caused by VZV infection, the diagnosis of RHS is delayed because of the late appearance of vesicles, interfering with early antiviral treatment. Nevertheless, PCR analysis of VZV DNA in saliva can help overcome this problem through early diagnosis and clinical evaluation of RHS. A limitation of this study is that the difference in clinical symptoms according to the VZV DNA level could not be analyzed because the DNA levels in saliva could not be measured quantitatively using our instrument. Moreover, accurate VZV DNA analysis was uncertain, as there were differences in the time of expression of facial palsy and the time of collecting saliva samples for VZV DNA testing among patients.

## 5. Conclusions

The initial HB grade was higher in the VZV DNA-positive group than that in the VZV DNA-negative group. Moreover, there were more patients with severe initial HB grades (V and VI) in the VZV DNA-positive group than in the VZV DNA-negative group. The ENoG values measured in the four facial muscles (frontalis, orbicularis oculi, nasalis, and orbicularis oris) were higher, indicating more severe clinical symptoms in the VZV DNA-positive group than the VZV DNA-negative group. However, there was no difference in the final HB grade, and hence no differences in prognosis and comorbidities between the two groups.

Thus, a VZV DNA detection test using saliva samples of patients with RHS can evaluate the clinical symptoms and provide early confirmation of VZV infection, enabling timely treatment through non-invasive strategies. 

## Figures and Tables

**Table 1:** Demographic information of patients with RHS according to presence or absence of salivary VZV DNA.

	Ramsay Hunt Syndrome		
Parameter	Saliva VZV DNA (+) (*n* = 72)	Saliva VZV DNA (−) (*n* = 28)	*p-*Value
Age, yr	53.08 ± 13.50	44.85 ± 21.16	0.065
Sex, *n* (%)			0.238
Men	21 (29.17)	12 (42.86)	
Women	51 (70.83)	16 (57.14)	
Direction, *n* (%)			0.238
Right	21 (29.17)	12 (42.86)	
Left	51 (70.83)	16 (57.14)	
Cranial polyneuropathy, *n* (%) (exclude CN VII or VIII)	3 (4.17)	0 (0.00)	0.273
Underlying disease			
Hypertension, *n* (%)	9 (12.50)	4 (14.28)	0.812
Diabetes, *n* (%)	9 (12.50)	0 (0.00)	0.058
Accompanying symptoms, *n* (%)			
Otalgia	54 (75.00)	24 (85.71)	0.246
Dizziness	21 (29.17)	8 (28.57)	0.953
Tinnitus	33 (45.83)	16 (57.14)	0.375
Hyperacusis	9 (12.5)	4 (14.29)	0.753
Hearing disturbance	23 (31.94)	5 (17.86)	0.159

**Table 2:** Number of patients with RHS depending on salivary VZV DNA and serum VZV IgM.

	Saliva VZV DNA (+)	Saliva VZV DNA (−)	Total
VZV IgM (+)	21	12	33
VZV IgM (−)	51	16	67
Total	72	28	100

**Table 3:** Differences in severe initial HB grade between the salivary VZV DNA-positive and negative groups.

	Initial HB Grade (Severe)			II–IV -> V,VI	
Parameter	II–IV	V,VI	*p-*Value	OR (95% CI)	*p-*Value
VZV DNA					
(−)	27 (96.43)	1 (3.57)		1	
(+)	54 (75.00)	18 (25.00)	0.014	34.023 (3.218–359.687)	0.003

**Table 4:** Differences in complete and satisfactory recovery between the salivary VZV DNA-positive and negative groups.

	Final HB Grade (Complete Recovery)			Final HB Grade (Satisfactory Recovery)		
Parameter	I	II–VI	*p-*Value	I,II	III–VI	*p-*Value
VZV DNA						
(−)	16 (57.14)	12 (42.86)		24 (58.71)	4 (14.29)	
(+)	33 (45.83)	39 (54.17)	0.31	54 (75.00)	18 (25.00)	0.246

**Table 5:** Differences between ENoG values measured in four facial muscles of VZV DNA-positive and negative groups.

Parameter	Saliva VZV DNA (+) (*n* = 72)	Saliva VZV DNA (−) (*n* = 28)	*p-*Value
ENoG			
Frontalis	78.85 ± 4.92	60.41 ± 3.08	0.003
Oculi	80.57 ± 3.38	58.66 ± 3.34	0.001
Nasalis	83.42 ± 3.54	60.58 ± 3.14	<0.001
Oris	85.71 ± 3.74	74.66 ± 3.05	0.037

## Data Availability

Data were collected during the study in Kyunghee Medical Center.
